# Editorial: Advancing physical activity behavior change: the potential of serious digital health technologies

**DOI:** 10.3389/fpsyg.2026.1929995

**Published:** 2026-07-29

**Authors:** Patrick Manser, Sascha Ketelhut, Eveline S. Graf, Elpidio Attoh-Mensah, Jean-Jacques Temprado, Louis Bherer

**Affiliations:** 1 Department of Health Sciences and Technology, ETH Zurich, Zurich, Switzerland; 2 Division of Physiotherapy, Department of Neurobiology, Care Sciences, and Society, Karolinska Institutet, Huddinge, Sweden; 3 Institute of Sport Science, University of Bern, Bern, Switzerland; 4 Exertion Games Lab, Department of Human-Centered Computing, Monash University, Melbourne, VIC, Australia; 5 School of Health Sciences, Institute of Physiotherapy, ZHAW Zurich University of Applied Sciences, Winterthur, Switzerland; 6 HAVAE UR 20217, University of Limoges, Limoges, France; 7 Institut des Sciences du Mouvement (ISM), UMR 7287, CNRS, Aix Marseille Université, Marseille, France; 8 Research Center and Centre ÉPIC, Montreal Heart Institute, Montreal, QC, Canada; 9 Department of Medicine, Faculty of Medicine, University of Montreal, Montreal, QC, Canada; 10 Research Center, Institut Universitaire de Gériatrie de Montréal, Montreal, QC, Canada

**Keywords:** behavior therapy, digital health, exercise, exergaming, health behavior, health promotion, healthy lifestyle, telemedicine

## 1 Tackling the public health issue of physical inactivity with digital health technologies (DHTs)

The Global Action Plan on Physical Activity (PA) targeted a 15% relative reduction in physical inactivity levels by 2030 (World Health Organization, 2019). Yet, despite widespread PA guidelines, the global prevalence of insufficient PA remains high and continues to rise (Strain et al., 2024). This persistent gap shows that generic recommendations alone are insufficient to promote lasting behavior change (Rhodes et al., 2019). Given the major individual and societal consequences of physical inactivity (Santos et al., 2023), this Research Topic addresses a central question: how and under what conditions can DHTs [i.e., an umbrella term for digital technologies applied in the context of health, including eHealth, mHealth, telehealth, and artificial intelligence (AI) (Herold et al., 2022)] that are purposefully designed to promote lasting health behavior change?

## 2 From habit formation to sustained behavior change: what DHTs Can (and cannot) do

A central message emerging from this Research Topic is that DHTs are best understood not as behavior change interventions in themselves, but as delivery platforms whose effectiveness depends on how they are designed, embedded, and supported.

### 2.1 Supporting health behavior habit formation

Baumann et al.'s bibliometric trend and network analysis of 2,981 articles anchors this Research Topic. They document the rapid expansion of mobile and wearable DHTs for PA promotion in the 2010s, reflecting advances in accessible PA self-monitoring. However, more structured support is required, as Adorni et al. demonstrated with appropriately timed, user-sensitive, and interactive communication grounded in the Health Action Process Approach (Schwarzer, 2008) that produced more sustained PA increases than self-monitoring. This finding aligns with broader evidence that DHTs can support habit formation at scale (Singh et al., 2024a; Mazeas et al., 2022)—provided they offer clear cues, immediate rewards, and repeated opportunities for action (Singh et al., 2024b), for which DHTs provide practical advantages via behavioral prompts, continuous feedback, and remote monitoring and tailoring.

### 2.2 Beyond habit formation—what drives lasting behavior change?

Initiating habit formation is a challenge. Sustaining improved health behavior is often even more challenging. Hart et al.'s protocol addresses this challenge methodologically through a two-stage randomization, allowing examination of both initial habit formation and the role of personalized digital support grounded in the Behavior Change Wheel (Michie et al., 2011) in maintaining PA after moderate-to-severe traumatic brain injury. For sustaining general PA over ≥1 year Li and Dong's findings on psychosocial mechanisms in adolescents reinforce that lasting behavior change cannot be reduced to behavioral prompts and rewards alone; programs must also foster perceived control, self-confidence, and social support.

Glatt et al.'s “*brain gym*” integrated these principles in a community-based senior living environment grounded in Self-Determination Theory (Ryan and Deci, 2000). By combining gamification, graded task progression, real-time feedback, and social facilitation, 67% met criteria for sustained adherence over 12 months. Although program frequency was low (< 1 session/week), this contribution is notable because long-term engagement remains a challenge and evidence on feasible community-based solutions is still rare (Manser et al., 2025).

### 2.3 Benefits beyond PA behavior?

DHT features can support health behavior change more broadly, as demonstrated by Liu et al. for supporting sustained self-management in chronic disease care with structured follow-up, personalized feedback, and closed-loop monitoring. Hao et al. further show that gamified DHTs (exergames) improve sleep quality, particularly among older people with health conditions and longer interventions—again emphasizing the importance of sustained implementation.

Clinical adoption of newer DHTs (e.g., extended reality exergames), however, has lagged, and integration into routine practice remains limited, as Baumann et al. showed. This highlights a central theme of this Research Topic: Theoretically grounded DHT choice and features can support behavior change and matter for workflow integration, alignment with reimbursement models, and safety requirements, but cannot substitute for structured support and social/professional guidance (Manser et al., 2025).

### 2.4 Theory and technology vs. human guidance—designing DHTs for healthcare implementation

Overcoming the evidence-to-practice gap of DHT implementation requires a balance between user-centered conceptualization, co-development, and evaluation with patient and public involvement and theoretically grounded attention to implementation conditions (Manser et al., 2026). Jakobsson et al.'s protocol illustrates these recommendations in practice by continuously accounting for future uptake during user-centered development and feasibility testing using a complexity-based framework for DHT implementation and sustainability (Greenhalgh et al., 2017).

Attoh-Mensah et al. complement these recommendations by showing how AI may augment clinical decision-making and improve access to more personalized care, while also highlighting barriers including cost, technological dependence, bias, privacy, and security. Baumann et al. caution that rigorous validation is needed to determine whether and how AI-enhanced coaching, digital twins, and related features may benefit health behavior change, as the evidence base remains thin.

## 3 Advancing from a fragmented field into a united scientific discipline to make public health impact

To conclude, the contributions share the aim of advancing health behavior, but span a breadth of DHTs, frameworks, populations, outcomes, and methodological approaches. This diversity reflects genuine scientific vitality but also signals a fragmented field that has not yet established shared constructs, research priorities or methodological standards.

This fragmentation is reflective of the wider literature, where overlapping concepts are pursued under separate labels with limited cross-referencing across these silos. This is especially prevalent for different DHT subconstructs (Herold et al., 2022) and gamified DHTs (i.e., exergames) that are referred to under various terms (Manser et al., 2025).

Therefore, the field's next maturation step may be less about continuing to produce isolated studies and more about conceptual and methodological consolidation across these silos. Efforts toward shared taxonomies (e.g., Manser et al., 2025; Torre and Temprado, 2022), consensus-based, unifying methodological guidelines (e.g., Manser et al., 2026), and systematic evidence mapping (Baumann et al. this Research Topic) are precisely the conceptual and methodological investments that can transform a productive but fragmented field into a mature and united scientific discipline.

We recommend distinguishing carefully what DHTs can and should do, and in which contexts they should be embedded to promote lasting health behavior change. The challenge ahead is not simply to develop more advanced DHTs, but to determine which technologies work for whom, under what conditions, through which mechanisms, and with what level of human and organizational support to effectively and sustainably embed them into tailored interventions, services, and everyday life to benefit all stakeholders. Only then can serious DHTs move from promising tools to reliable contributors to sustained health behavior change and public health impact.
